# Malaria among children living in flood-prone villages in the Kasese District of Western Uganda: prevalence and risk factors from a cross-sectional study

**DOI:** 10.21203/rs.3.rs-10535941/v1

**Published:** 2026-10-04

**Authors:** Rogers Kalyetsi, Ronnie Ndizeye, Michael Reiskind, Raquel Reyes, Kaylin Lewandowski, Mbusa Rapheal, Abel Bwambale, Baguma Emmanuel, Liao Huipeng, Dana Anne Giandomenico, Bonnie E Shook-Sa, Moses Ntaro, Peter Chris Kawungezi, Hope Mudondo, Charles Nkubi Bagenda, Paul E. Alele, Jerome Kabakyenga, Gertrude Kiwanuka, Edgar M. Mulogo, Ross M. Boyce

**Affiliations:** Mbarara University of Science and Technology; Mbarara University of Science and Technology; North Carolina State University; University of North Carolina at Chapel Hill; North Carolina State University; Mbarara University of Science and Technology; Mbarara University of Science and Technology; Mbarara University of Science and Technology; University of North Carolina at Chapel Hill; University of North Carolina at Chapel Hill; University of North Carolina at Chapel Hill; Mbarara University of Science and Technology; Mbarara University of Science and Technology; Mbarara University of Science and Technology; Mbarara University of Science and Technology; Mbarara University of Science and Technology; Mbarara University of Science and Technology; Mbarara University of Science and Technology; Mbarara University of Science and Technology; University of North Carolina at Chapel Hill

**Keywords:** Children, Flood-prone villages, Malaria, Plasmodium falciparum, Positivity, Uganda

## Abstract

**Background:**

*Plasmodium falciparum* infections pose significant health and economic challenges in Uganda. This study evaluated the prevalence of *P. falciparum* malaria among children in flood-prone villages in Kasese District, Western Uganda, and examined associated factors.

**Methods:**

A total of 7,987 children 2–12 years of age from 3,702 unique households consented to participate. Demographic data and malaria prevention and treatment behaviours were documented. Capillary blood samples were aseptically collected and tested for *P. falciparum* circulating HRP1&2 using mRDT. Chi-square or Fisher’s exact tests were used to compare malaria prevalence, while logistic regression identified factors associated with malaria positivity. Generalized estimating equations were used to estimate associations between malaria positivity and factors including age, sex, terrain, LLIN usage.

**Results:**

The *P. falciparum* positivity rate was 3.3% (267 of 7.987; 95% CI: 3.0–3.8) by RDT.. Among those who had fever (temp ≥ 37.5°C) at the time of the visit, the PfPR was 10.1% (40/396), compared to 3.0% (227/7,591) among those without a fever. Of the 3,702 households, 213 (5.75%, 95% CI 5.05–6.55) had at least one positive malaria case. The positivity rates varied with sub-county of residence (Kyarumba 8.0% and Karusandara 10.0%); sex (female 2.7% vs. 4.1% males); LLIN use (4.2% No vs. 2.0% yes); and altitude (low 3.8% vs. high 0.5%). Individual positivity clustered at the sub-villages was independently associated with 5–12 years (aOR 2.15, 95% CI 1.37, 3.39; p < 0.001), male sex (aOR 1.46, 95% CI 1.18, 1.81; p = 0.001), fever (aOR 3.08, 95% CI 2.00, 4.74; p < 0.001) and use of LLIN a previous night (aOR 0.57, 95% CI 0.43, 0.74; p < 0.001). The household positivity was independently associated with LLIN ownership status (aOR 0.53, 95% CI 0.39, 0.72; p < 0.001), surface water for domestic use (aOR 5.17, 95% CI 3.60, 7.42; p < 0.001), and > 6 occupants in the household (aOR 2.7, 95% CI 1.62, 4.63; p < 0.001).

**Conclusion:**

The findings illustrate ongoing malaria transmission. Household clustering with malaria highlights the presence of hotspots in Kyarumba and Karusandara sub-counties. Targeted campaigns to enhance household ownership and usage of LLINs could further protect against malaria following severe flooding events.

## Background

Uganda is among the countries experiencing the greatest burden of malaria, contributing ~ 5% of cases and ~ 3% of deaths globally [1]. Malaria is endemic in more than 90% of Uganda and accounts for 30 − 50% of outpatient visits[1] and half of paediatric inpatient deaths [2]. The disease poses a substantial burden on the health, education, and economic improvement of Ugandans, with those most vulnerable to developing severe disease including children under five years of age [3], elementary school children [4], and pregnant women [2, 5].

The Kasese District in western Uganda experiences perennial, seasonal rainfall with annual flood events of varying severity along the major rivers that flow down from the Rwenzori Mountains [6–10]. Flooding results in the expansion of surface water with increases in vector habitat, abundance, and consequent malaria risk [10, 11]. The floods that occur in the district of Kasese are somewhat predictable, taking place in early to mid-May, often with subsequent malaria epidemics. However, despite the predictability of floods and related negative effects, estimates of malaria burden among vulnerable communities following flood episodes are limited [9, 12, 13]. The rationale of this pre-trial study was to map malaria hotspots, stratify community risk, and inform trial design, stratified randomization, and public health actions. We hypothesized that *Plasmodium falciparum* positivity rates vary across different study villages prone to floods in Kasese district. This study evaluated the prevalence of *P. falciparum* malaria among children aged ≤ 12 years in Kasese district and identified demographic factors associated with malaria positivity rate in this population. The findings provide vital data on malaria burden in this high-risk, flood-prone setting, informing targeted intervention efforts in addition to informing a cluster-randomized controlled trial design.

## Methods

### Study design

This was a baseline cross-sectional study in 160 sub-villages in preparation for a cluster- randomized controlled trial of post-flooding malaria control (NCT06870344). A sub-village (approximately 44.6 households, 197 people) was under the supervision of a trained village health worker officially designated as a Village Health Team (VHT) member. The study involved sensitizing sub-county and village leaders about its objectives through meetings and radio talks. The VHTs mobilized households with children aged 2 to 12 for the study. Children from a total of 160 sub-villages that reported recent floods were selected to participate in the study. On the survey day, study personnel, including lab technologists and data clerks, visited homes with support from VHTs. Research assistants explained the study to adults in the local language, Lukhonzo, explaining the risks and benefits for the intended participants, children aged 2 to 12. Consent was obtained from parents/ guardians, and in addition, assent was obtained from children aged 8–12 years old.

### Study settings and period

The study was conducted in Kasese District of south-western Uganda between 19th February and 22nd May 2025. The district covers approximately 2,700 km^2^, characterized by a rural landscape, deep river valleys, and steep hillsides with elevations of up to 5,000 m. The equatorial climate allows for year-round malaria transmission, with peaks in January and July [10, 14]. Within Kasese, the study area included twelve (12) administrative units at sub- county level within the catchments of the Mubuku, Nyamwamba and Nyamugasani rivers (Fig. 1).

### Participants and recruitment

Village Health Team members mobilized households with children aged 2–12 years. Sub-villages were purposively selected because they were located within flood-prone river catchments and represented areas of moderate-to-high malaria transmission. Household eligibility was based on participants being permanent residents, with a reported history of being affected by flooding in a previous year, and having at least one child aged 2–12 years.

On survey days, trained study personnel visited participating households with support from the Village Health Teams. Research assistants explained the study in Lukhonzo, including the study procedures, potential risks, and benefits. Eligibility criteria for study participants included being aged 2–12 years, residing permanently in a selected flood-prone sub-village, having a reported history of household exposure to flooding in the preceding year, and having a parent or legal guardian able and willing to provide written informed consent. Children aged 8–12 years were additionally required to provide assent.

### Variables

The primary individual-level outcome was malaria positivity, defined as a positive SD Bioline Malaria Ag *P. falciparum* rapid diagnostic test result. The household-level outcome was the presence of at least one mRDT-positive child in the household.

Prespecified individual-level explanatory variables included age, sex, axillary temperature, educational level, sub-county of residence, river catchment, distance from the river, altitude, LLIN use the previous night, LLIN condition, and LLIN age. Household-level explanatory variables included LLIN ownership, flooding history in the previous year, main household water source, location of the water source, sanitation facility, and household size.

### Data sources and measurements

After obtaining written consent, household geo-location (GPS coordinates) and demographic data were collected using modified questions from the Uganda Malaria Indicator Survey in a cloud database [15]. Children’s forehead temperatures were recorded, and capillary blood samples were taken for malaria testing using a rapid diagnostic test (SD Bioline Malaria Ag *P. falciparum*, Abbott Laboratories, USA)[15].

Household geographical coordinates were recorded using GPS. Altitude and river-catchment variables were derived from the recorded household locations. Household and participant characteristics were collected using questions adapted from the Uganda Malaria Indicator Survey and entered directly into REDCap.

### Efforts to address potential bias

The study used a census approach within selected sub-villages to reduce participant-selection error. Standardized questionnaires, trained field teams, uniform temperature measurement, and a single mRDT product were used to improve measurement consistency. Generalized estimating equations with robust standard errors were used to account for clustering at the sub-village level.

### Study size

A census approach was used. All eligible children available in the 160 selected sub-villages during the survey period were invited to participate, yielding 7,987 participants from 3,702 households. The census was intended to establish a comprehensive baseline sampling frame for the parent trial and to estimate malaria positivity across the selected flood-prone communities

### Handling of quantitative variables

Age and axillary body temperature were summarized using medians and interquartile ranges and were also categorized for analysis. Age was categorized as < 5 years and 5–12 years. Axillary temperature was categorized as < 37.5°C and ≥ 37.5°C. Altitude was categorized as low (< 1,000 m), moderate (1,000–1,500 m), and high (> 1,500 m). Distance from the river was categorized as near (< 2 km) and far (≥ 2 km).

### Statistical analysis

Data analysis was conducted using STATA software (release 17). Malaria positivity was calculated as a proportion of positive tests, with results shown as frequencies and percentages. A chi-squared or Fisher’s exact test compared malaria prevalence across categories of independent variables. Generalized estimating equations (GEE) were used to estimate associations between malaria positivity and factors including age, sex, terrain, LLIN usage, flooding history, and water source. Bivariate and multivariate models were fitted assuming a binomial distribution and a logit link for estimation of crude and adjusted odds ratios, respectively. These models accounted for clustering at the sub-village level using an exchangeable working correlation structure. Robust (sandwich) variance estimates were used to construct 95% confidence intervals and to conduct hypothesis testing, with a 0.05 significance level. No adjustments were made for multiple comparisons.

### Ethical considerations

All protocols were reviewed and approved by the Mbarara University of Science and Technology Research Ethics Committee (MUST-2024-1538), and the Uganda National Council for Science and Technology (HS4978ES). Written informed consent was obtained from parents or legal guardians, and written assent was obtained from children aged 8–12 years. Participant confidentiality was maintained throughout data collection and analysis by using identification codes instead of names.

## Results

### Participant flow

Between February and May 2025, 7,987 eligible children from 3,702 households from 160 sub-villages that provided consent were included in the analysis (Fig. 2).

### Descriptive characteristics of study participants

Between 19th February and 22nd May 2025, 7,987 eligible participants were recruited from 3,702 households. Among the participants, 51.2% were female, and the median age was 6 (Table 1). Household ownership of at least one LLIN was 49.5%, LLIN utilization the previous night was 37.6%, and 74.9% of LLINs used were ≥ 2 years old. A total of 267 (3.3%, 95% CI: 3.0, 3.8) participants tested positive for malaria by RDT. Malaria positivity among those with fever (temp ≥ 37.5°C) was 10.1% (40/396) compared to 3.0% (227/7591) among those with normal body temperature. A total of 213 households had at least one child who tested positive (5.8%; 95% CI: 5.05, 6.55).

### Unadjusted associations

Our bivariate analysis of associations between malaria positivity and risk factors for individuals showed a number of significant demographic and geographic associations among sub-village clusters. Malaria positivity was higher among males compared to females (4.1% to 2.7%), higher in the 5–12 age group compared to under 5 years (4.0% to 2.1%), and comparing those with body temperature ≥ 37.5°C to those without fever (10.1% to 3.0%) (Table 2). Positivity was higher among children from Karusandara (10%) and Kyarumba (8.0%) sub-counties, relative to other sub-counties. Geography was also important, with higher malaria prevalence in participants from the Mubuku River catchment (3.9%) and Nyamugasani River catchment (3.9%) relative to the Nyamwamba. Similarly, malaria prevalence varied with altitude, with those at a low altitude (less than 1000m) having a malaria prevalence of 3.8% compared to 0.5% at higher altitude.

In addition to comparing individual participant risk factors, we also evaluate the risk of a household having at least one positive malaria case relative to a number of variables. The prevalence rate at the household level varied with LLIN ownership status, with 7.6% among households that did not own any compared to 3.8% among those that owned at least a net. History of flooding significantly increased the risk of malaria, with 7.4% of children from household reported history of flooding compared positive for malaria, compared to 4.9% who did not experience flooding in the previous year. Similarly, the positivity rate was higher where the water source water borehole 14.2% or surface water 11.4% compared to 2.2% for piped water (Table 3).

### Adjusted associations

Examining associated factors in a multivariate logistic regression for individual participants, after adjusting for cofounders, malaria positivity was independently associated with 5–12 age group of the child (aOR 2.15, 95% CI 1.37, 3.39; p = 0.001), male gender (aOR 1.46, 95% CI 1.18, 1.81; p = 0.001), fever (temp ≥ 37.5°C) (aOR 3.08, 95% CI 2.00, 4.74; p < 0.001), and not using LLIN previous night before the study (aOR 0.57, 95% CI 0.43, 0.74; p < 0.001) (Table 4). At the household level, our multivariate approach found that using a surface water source for domestic use (aOR 5.17, 95%CI 3.60, 7.42; p < 0.001) and > 6 occupants in a household (aOR 2.74, 95%CI 1.62, 4.63; p < 0.001) were independently associated with malaria positivity (Table 5).

## Discussion

This was a cross-sectional study where 7,987 children aged 2–12 years were recruited from flood-prone villages in the Kasese district. Peripheral blood samples were collected aseptically and tested for *P. falciparum* circulating antigens between February and May 2025. Malaria positivity was associated with demographic and geographic factors, including a history of flooding and living in areas prone to seasonal, riverine floods. Nevertheless, our overall estimate of malaria prevalence was well below previous estimates [10, 16].

Our findings are in agreement with other recent studies in Uganda demonstrating decreased malaria prevalence after the most recent LLIN campaign [17–20]. These observations could be attributed to variations in seasonal transmission peaks[10, 21–23], low vector abundance in dry seasons[24], increased LLIN coverage[25–27], and low sensitivity of mRDT in endemic settings [28, 29].

Another curious result was the higher prevalence levels of malaria in children aged 5–12 years relative to children under 5 years [30–33]. The reason for this finding may be that in higher transmission settings, it is possible for the infection to overpower the immunity of children [31, 34], and they less frequently utilize LLINs and have increased exposure to vectors because of their involvement in outdoor activities like fetching water away from their homes [35]. In addition, decreased transmission of pathogens may shift populations towards an endemic stability, in which the average age of first infection is older[36]. Our findings are contrary to what was observed in other studies in endemic areas that established that children below five years are at higher morbidity and mortality risk and other severe outcomes of malaria infections [37, 38]. This could be because of the weak immunity that is overwhelmed by intense malaria transmission [34, 37, 38]. The higher prevalence of malaria among primary school children may indicate active transmission in the community and schools due to exposure to outside mosquito bites. These findings relate to the literature from studies in Kenyan lowlands and Rwandan highlands [4, 35]. Targeted school-based interventions like classroom screening of ventilators and chemoprophylaxis may be important to slow transmission in this age group.

The data indicate distinct geographical patterns in malaria transmission across various sub-counties and river basins, highlighting Karusandara and Kyarumba sub-counties as malaria transmission hot zones, likely due to local environmental, infrastructural, and geographical factors [10, 39]. These findings could be attributable to environmental factors, including many vector breeding sites, outdoor and indoor exposures to mosquito bites [40, 41], low LLIN ownership and utilization, and dependence on surface water sources located far from the households which are positively associated with malaria positivity and transmission. Karusandara and Kyarumba sub-counties are also located within the lowlands of the Kasese District that experience severe effects of flooding, poor drainage that results in large areas of suitable larval habitat, and close proximity to flood-prone rivers [9, 10]. These observations are in agreement with the literature, indicating that flood-prone rivers are associated with increased malaria risk [10]. In addition, high malaria risk aggregated along the banks of the rivers post-flood episodes corresponds with other literature in similar regions [42]. Similarly, residences within close proximity to flood-prone rivers and with standing water in the vicinity also had higher malaria positivity rates [43, 44].

Children from low-lying areas had a higher 3.8% prevalence, seven times higher compared to those from higher elevations (0.5%). Areas of moderate altitude (3.2%) were of intermediate risk. This highlights the importance of altitude protection in regions where malaria is widespread and affects vulnerable children. This is because low-lying areas may easily become a permanent breeding ground for *Anopheles*, such as after a flood [9, 10]. Analysis of the findings clearly shows a wide difference in areas of malaria transmission and also a distinct difference in geographical factors influencing these flood-vulnerable areas [39, 45]. These results are in line with other related studies [46, 47]. By contrast, the 2013 floods in the Kasese area of the Ugandan highlands showed lower post-flood risk with increasing altitude, consistent with the impact of shared topography and proximity to rivers [10]. There was a negative altitude and incidence correlation in different studies in Rwanda and Uganda [47, 48]. These differences could be attributed to variations in land use changes [47, 48].

The positivity in household malaria infections was significantly associated with a history of flooding, the use of surface and/or borehole water, and collecting water away from home. Such modifiable environmental variables increase the prevalence of mosquito-breeding and children’s bite exposure during flood episodes, post-floods, and while collecting water [49, 50]. Interventions such as increasing accessibility to piped water, constructing flood-resistant drains, protecting water sources, and distributing larvicides could help prevent cases of malaria. Our results agree with other post-flood investigations that identified a greater positivity of malaria closer to riverbanks [10], and with unimproved and surface water sources [50, 51]. These findings are consistent with previous studies from Rwanda and Kenya [35, 38]. By contrast, literature from other studies [49, 51, 52] reported a negative correlation between flooding and malaria, which may be indicative of dissimilarities in specific conditions we find in Kasese District in terms of elevation change, use of LLINs, or other factors.

### Strength and limitations

The study included a large community- based sample from numerous flood-prone villages and used analytical methods that accounted for clustering at the sub-village level.

The cross-sectional nature of the design used makes the interpretation of the relationship between observed malaria positivity and associated factors difficult, as these may be the result of confounding variables we did not measure or coincidence rather than true causal links. Future studies can consider analysing trends in exposure or outcomes across different time periods.

The mRDT employed in this study could have missed identifying infections with diminished parasite loads in the bloodstream, and its inadequate sensitivity to detect infections from other *Plasmodium* species may have resulted in reduced case detection rates[53]. The generalization of our study findings is limited, even to the whole Kasese District as the study focused on only flood-prone areas.

### Generalizability

The findings are most applicable to children aged 2–12 years living in flood-prone communities of Kasese District during the February–May 2025 survey period. Generalization to the entire district, non-flood-prone communities, other seasons, adults, or settings with different malaria transmission intensity and vector-control coverage should be made cautiously.

## Conclusions

Established geographic factors, including exposure to seasonal flooding were associated with P. falciparum prevalence among children in western Uganda. Household clustering with malaria highlights the presence of hotspots, most especially in Kyarumba and Karusandara sub-counties. Targeted campaigns to increase household ownership of LLINs and use, increasing accessibility to piped water, and sensitizing communities about care for LLINs and repair could lengthen useful life, and together with promoting sleeping under the net in high malaria transmission villages, could be protective following flooding events.

## Supplementary Material

This is a list of supplementary files associated with this preprint. Click to download.

- ChecklistcrosssectionalJuly2026.docx

## Figures and Tables

**Figure 1:**
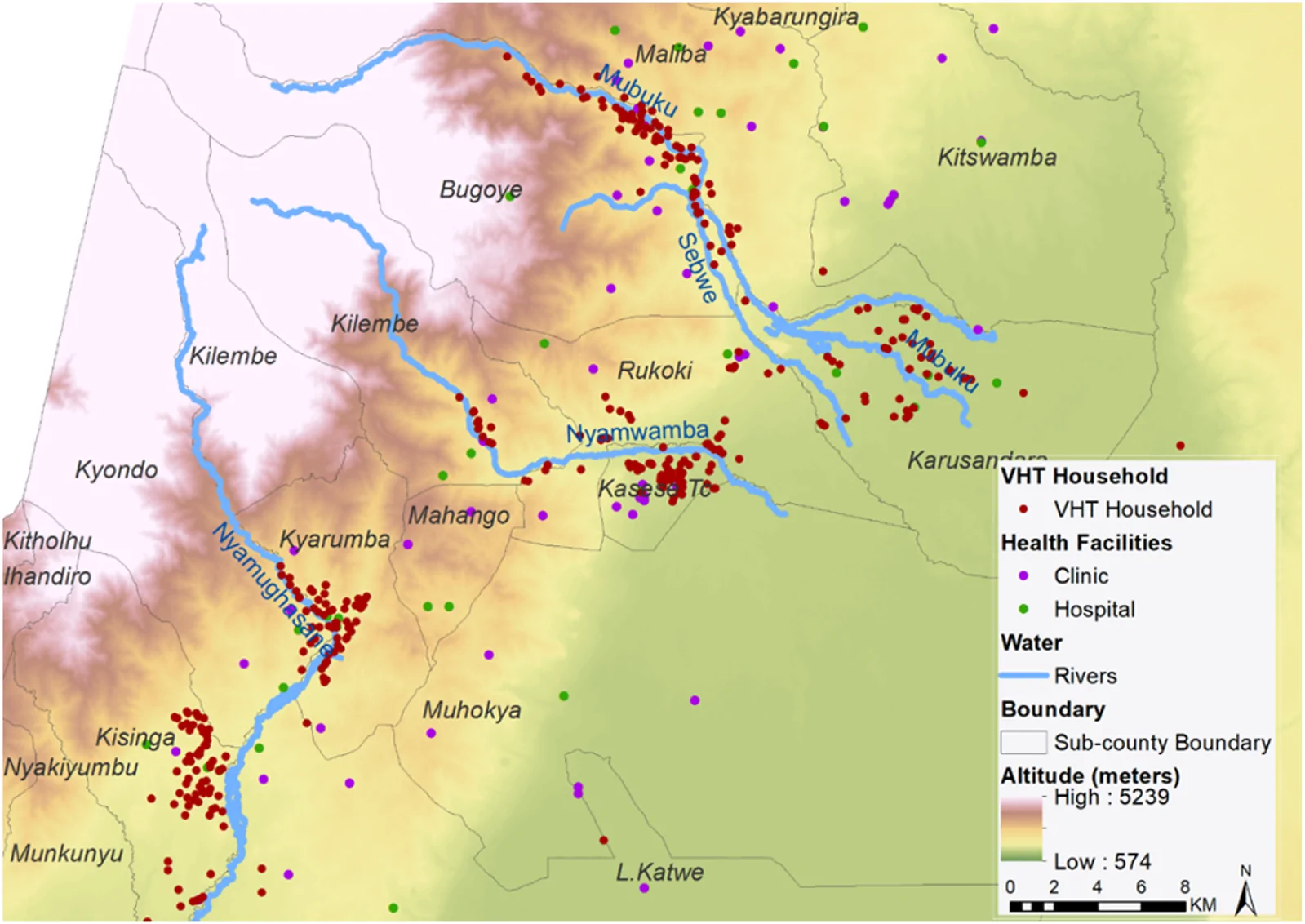
Map showing the location of the study settings, Kasese district

**Figure 2:**
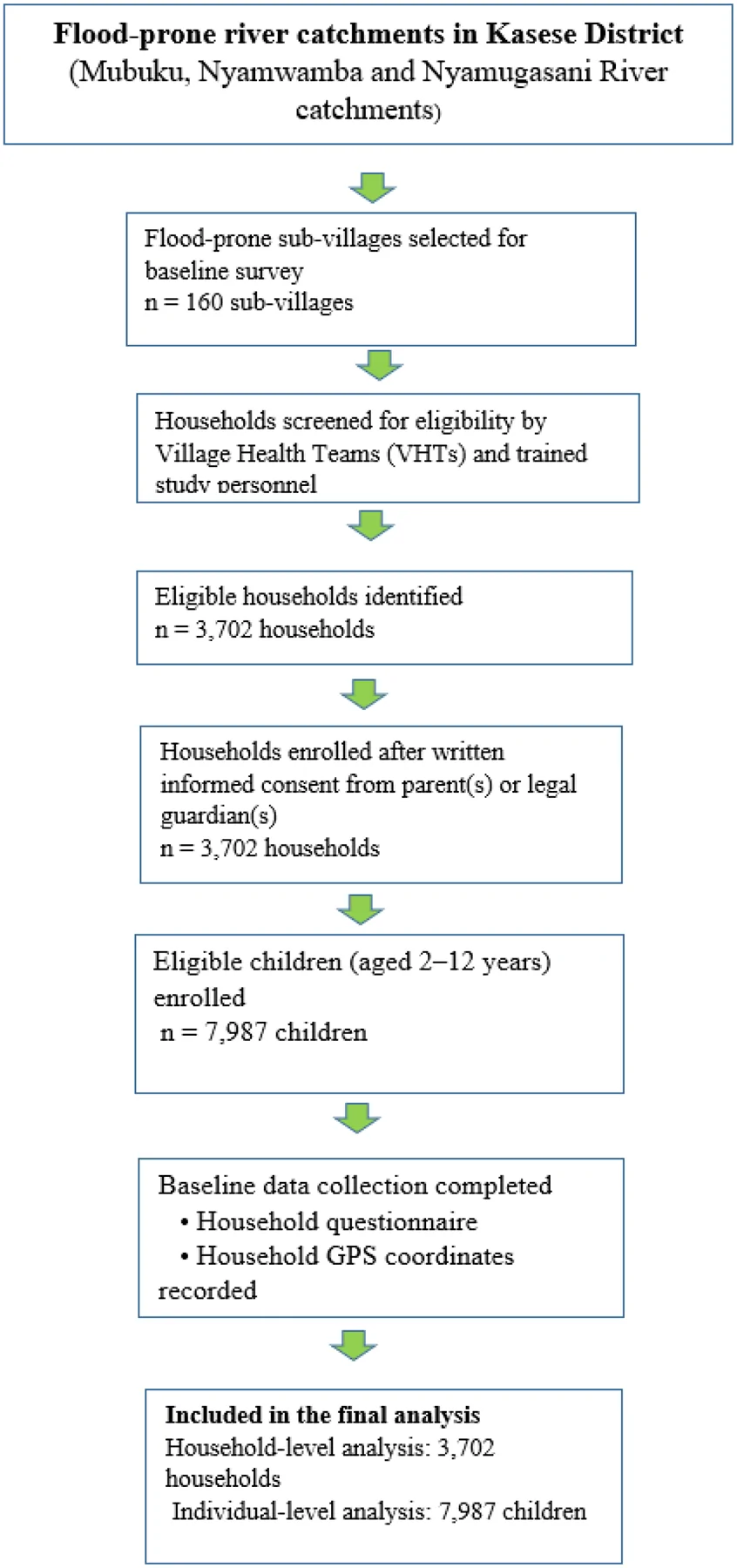
Study participant flow diagram

**Table 1:** Demographic and household characteristics of study participants across study area

Study variable	Total
	N = 7,987
Age (Years): Median (IQR)	6 (4–9)
Age group (Years)	
<5	2,626 (32.9%)
5–12	5,361 (67.1%)
Sex of study participant	
Female	4,092 (51.2%)
Male	3,895 (48.8%)
Axillary Temperature(°C): Median (IQR)	36.6 (36.2–36.9)
Axillary temperature (°C)	
<37.5	7,591 (95.0%)
≥37.5	396 (5.0%)
Level of formal education	
No school	1,579 (19.8%)
Nursery school	2,357 (29.5%)
Primary school	4,051 (50.7%)
Place of residence (Sub-county)	
Bugoye	521 (6.5%)
Bulembya	396 (5.0%)
Ibanda	234 (2.9%)
Ibanda Town Council	774 (9.7%)
Karusandara	944 (11.8%)
Kasese municipality	411 (5.1%)
Kisinga	718 (9.0%)
Kisinga Town Council	766 (9.6%)
Kyarumba	922 (11.5%)
Kyarumba Town council	543 (6.8%)
Maliba Town Council	101 (1.3%)
Mubuku Town Council	79 (1.0%)
Nyamwamba Division	1,578 (19.8%)
Catchment area (River)	
Mubuku	2,723 (34.1%)
Nyamugasani	2,949 (36.9%)
Nyamwamba	2,315 (29.0%)
Slept in LLIN previous night	
No	4,980 (62.4%)
Yes	3,007 (37.6%)
Condition of LLIN (Holes)	
No	1,157 (38.5%)
Yes	1,850 (61.5%)
Age LLIN (Years)	
< 1	756 (25.1%)
1–2	1,874 (62.3%)
> 2	377 (12.5%)

**Table 2:** Distribution of individual-level malaria positivity across participants’ characteristics

Variable	Malaria results		p-value
	Negative N = 7,720 96.7% (95% CI: 96.2, 97.0)	Positive N = 267 3.3% (95% CI: 3.0, 3.8)	
Age (Years): Median (IQR)	6 (4–9)	7 (5–10)	<0.001
Age group (Years)			<0.001
< 5	2,572(97.9%)	54 (2.1%)	
5–12	5,148 (96.0%)	213 (4.0%)	
Sex of study participant			<0.001
Female	3,983 (97.3%)	109 (2.7%)	
Male	3,737 (95.9%)	158 (4.1%)	
Temperature (°C): Median (IQR)	36.5 (36.2–36.9)	36.8 (36.4–37.2)	<0.001
Temperature (°C)			<0.001
<37.5	7,364 (97.0%)	227 (3.0%)	
≥37.5	356 (89.9%)	40 (10.1%)	
Level of formal education			0.21
No school	1,537 (97.3%)	42 (2.7%)	
Nursery school	2,278 (96.6%)	79 (3.4%)	
Primary school	3,905 (96.4%)	146 (3.6%)	
Place of residence (Sub-county)			<0.001
Bugoye	505 (96.9%)	16 (3.1%)	
Bulembya	394 (99.5%)	2 (0.5%)	
Ibanda	234 (100.0%)	0 (0.0%)	
Ibanda TC	770 (99.5%)	4 (0.5%)	
Karusandara	850 (90.0%)	94 (10.0%)	
Kasese municipality	409 (99.5%)	2 (0.5%)	
Kisinga	708 (98.6%)	10 (1.4%)	
Kisinga TC	746 (97.4%)	20 (2.6%)	
Kyarumba	848 (92.0%)	74 (8.0%)	
Kyarumba town council	531 (97.8%)	12 (2.2%)	
Maliba T C	96 (95.0%)	5 (5.0%)	
Mubuku TC	77 (97.5%)	2 (2.5%)	
Nyamwamba Division	1,552 (98.4%)	26 (1.6%)	
Catchment area (River)			<0.001
Mubuku	2,617 (96.1%)	106 (3.9%)	
Nyamugasani	2,833 (96.1%)	116 (3.9%)	
Nyamwamba	2,270 (98.1%)	45 (1.9%)	
Slept in LLINs the previous night			<0.001
No	4,773 (95.8%)	207 (4.2%)	
Yes	2,947 (98.0%)	60 (2.0%)	
Condition of LLIN (holes)			0.41
No	1,137 (98.3%)	20 (1.7%)	
Yes	1,810 (97.8%)	40 (2.2%)	
Age LLIN (years)			0.16
< 1	747 (98.8%)	9 (1.2%)	
1–2	1,833 (97.8%)	41 2.2%)	
>2	367 (97.3%)	10 (2.7%)	
Distance sub-county from river bank			0.15
Near River (< 2 km)	3,555 (96.3%)	135 (3.7%)	
Far from liver (> 2 km)	4,165 (96.9%)	132 (3.1%)	
Altitude			0.002
Low (< 1,000m)	3,419 (96.2%)	136 (3.8%)	
Moderate (1,000m – 1,500m)	3,907 (96.8%)	129 (3.2%)	
High (> 1,500m)	394 (99.5%)	2 (0.5%)	

**Table 3:** Distribution of household- level malaria positivity across household characteristics

Variable	Negative	Positive	p-value
	N = 3,489	N = 213	
Ownership of LLINs			<0.001
No	1,728 (92.4%)	143 (7.6%)	
Yes	1,761 (96.2%)	70 (3.8%)	
History flooding over the past year			0.002
No	2,322 (95.1%)	120 (4.9%)	
Yes	1,167 (92.6%)	93 (7.4%)	
Main source of water for the household			<0.001
Piped water	2,292 (97.8%)	52 (2.2%)	
Surface water (river, lake, stream, irrigation channel)	771 (88.6%)	99 (11.4%)	
Tube well or borehole	356 (85.8%)	59 (14.2%)	
Water from the spring	70 (95.9%)	3 (4.1%)	
Location water source			<0.001
Elsewhere	2,498 (93.1%)	184 (6.9%)	
In one's own dwelling	991 (97.2%)	29 (2.8%)	
Type of latrine/ toilet facility			0.57
Composting toilet	32 (97.0%)	1 (3.0%)	
No facility/bush	8 (88.9%)	1 (11.1%)	
Pit Latrine	3,449 (94.2%)	211 (5.8%)	
Number of children in the home household			<0.001
≥2	1,546 (96.1%)	62 (3.9%)	
3–5	1,733 (93.1%)	129 (6.9%)	
≤6	210 (90.5%)	22 (9.5%)	

**Table 4:** Individual-level factors associated with malaria positivity, accounting for clustering at sub-village level

Variable	Bivariate		Multivariate	
	cOR (95% CI)	p-value	aOR(95%CI)	p-value
Age group (Years)				
<5	1.00		1.00	
5–12	1.73 (1.29, 2.32)	<0.001	2.15 (1.37, 3.39)	0.001
Sex				
Female	1.00		1.00	
Male	1.48 (1.19, 1.84)	<0.001	1.46 (1.18, 1.81)	0.001
Temperature (°C)				
<37.5	1.00		1.00	
>37.5	3.19 (2.09, 4.85)	<0.001	3.08(2.00, 4.74)	<0.001
Level of formal education				
No school	1.00		1.00	
Nursery school	1.28 (0.94, 1.756)	0.115	0.84(0.57, 1.24)	0.378
Primary school	1.44 (1.05, 1.97)	0.022	0.77(0.51, 1.15)	0.202
Distance of the sub-county from the river				
Near	1.00		1.00	
Far	0.72 (0.38, 1.36)	0.306	1.0(0.56, 1.77)	0.336
Altitude (m)				
Low (<1,000)	1.00		1.00	
Moderate (1,000–1,500)	0.67 (0.34, 1.28)	0.221	0.52(0.27, 0.99)	<0.045
High (>1,500)	0.120 (0.03,0.42)	0.001	0.27 (0.06, 1.17)	0.079
Catchment area (River)				
Nyamwamba	1.00		1.00	
Nyamugasani	2.09 (0.93, 4.66)	0.073	2.28(0.97, 5.35)	0.059
Mubuku	2.20(0.98, 4.92)	<0.056	1.75(0.69, 4.49)	0.239
Slept under LLINs the previous night				
Yes	1.00		1.00	
No	0.58 (0.44, 0.77)	<0.001	0.57(0.43, 0.74)	<0.001
Condition of LLIN (holes)				
No	1.00		-	
Yes	1.02 (0.64, 1.64)	0.920	-	
Age LLIN (years)				
< 1	1.00		-	
1–2	1.94 (0.93, 4.06)	0.075	-	
>2	1.79 (0.62, 5.17)	0.079	-	

cOR = crude odds ratio, aOR = adjusted odds ratio, CI = confidence interval.

**Table 5:** Household-level factors associated with at least one malaria-positive child.

Variable	Bivariate		Multivariate	
	cOR(95%CI)	p-value	aOR (95% CI)	p-value
Ownership of LLINs				
Yes	1.00		1.00	
No	0.48(0.36,0.644)	< 0.001	0.53(0.39,0.72)	< 0.001
History flooding Over the past year				
No	1.00		1.00	
Yes	1.54 (1.17, 2.04)	0.002	1.21(0.90,1.64)	0.208
Main source of water for the household				
Piped water	1.00		1.00	
Surface water (river, lake, stream, irrigation channel)	5.66(4.01, 7.99)	< 0.001	5.17(3.60, 7.42)	< 0.001
Tube well or borehole	7.30(4.95,10.78)	< 0.001	6.18(4.06 9.40)	< 0.001
Water from the spring	1.89(0.58, 6.195	0.294	1.91(0.58, 6.32	0.288
Location water source				
Elsewhere	1.00		1.00	
In one’s own dwelling	0.40(0.27, 0.59)	< 0.001	0.76(0.50,1.16)	0.204
Type of latrine/ toilet facility				
No facility/bush	1.00			
Composting toilet	0.25 (0.014, 4.44)	0.345	-	
Pit Latrine	0.49 (0.02, 1.00)	0.501	-	
Number of children in the home household				
<2	1.00		1.00	
3–5	1.86(1.36, 2.53)	< 0.001	1.94(1.41, 2.67)	< 0.001
>6	2.61(1.57, 4.33)	< 0.001	2.74(1.62, 4.63)	< 0.001

## Data Availability

The individual data that supports the results will be shared following publication provided the investigator who proposes to use the data has approval from an Institutional Review Board (IRB), Independent Ethics Committee (IEC), or Research Ethics Board (REB), as applicable, and executes a data use/sharing agreement with UNC.

## Abbreviations

ACT: Artemisinin-based Combination therapy
aOR: adjusted odds ratio
CI: Confidence Intervals
cOR: crude odds ratio
HRP 1 & 2: Histidine Rich Proteins 1 & 2
LLINs: Long-lasting insecticidal nets
Pf mRDT: *Plasmodium falciparum* malaria Rapid Diagnostic Test
Pf: Plasmodium falciparum
VHTs: Village Health Teams
VIF: Variance Inflation Factor
